# Cancer mortality in East and Southeast Asian migrants to New South Wales, Australia, 1975–1995

**DOI:** 10.1038/sj.bjc.6690205

**Published:** 1999-03

**Authors:** M McCredie, S Williams, M Coates

**Affiliations:** Cancer Epidemiology Research Unit, New South Wales Cancer Council, Kings Cross, NSW, 2011, Australia; Department of Preventive and Social Medicine, University of Otago, PO Box 913, Dunedin, New Zealand

**Keywords:** cancer, mortality, migrants, Australia, Asia, duration of residence

## Abstract

Routinely collected data for New South Wales were used to analyse cancer mortality in migrants born in East or Southeast Asia according to duration of residence in Australia. A case-control approach compared deaths from cancer at particular sites with deaths from all other cancers, adjusting for age, sex and calendar period. Compared with the Australian-born, these Asian migrants had a 30-fold higher risk of dying from nasopharyngeal cancer in the first 2 decades of residence, falling to ninefold after 30 years, and for deaths from liver cancer, a 12-fold risk in the first 2 decades, falling to threefold after 30 years. The initial lower risk from colorectal, breast or prostate cancers later converged towards the Australian-born level, the change being apparent in the third decade after migration. The relative risk of dying from lung cancer among these Asian migrants was above unity for each category of duration of stay for women, but at or below unity for men, with no trend in risk over time. An environmental or lifestyle influence for nasopharyngeal and liver cancers is suggested as well as for cancers of colon/rectum, breast and prostate. © 1999 Cancer Research Campaign


*British Journal of Cancer* (1999) **79**(7/8), 1277–1282
© 1999 Cancer Research Campaign
Article no. bjoc.1998.0205

### Cancer mortality in East and Southeast Asian migrants to New South Wales, Australia, 1975–1995

**M McCredie[1,2], S Williams[2] and M Coates[1]**

[1]Cancer Epidemiology Research Unit, New South Wales Cancer Council, PO Box 572, Kings Cross, NSW 2011, Australia; [2]Department of Preventive and Social Medicine, University of Otago, PO Box 913, Dunedin, New Zealand

**Summary** Routinely collected data for New South Wales were used to analyse cancer mortality in migrants born in East or Southeast Asia according to duration of residence in Australia. A case-control approach compared deaths from cancer at particular sites with deaths from all other cancers, adjusting for age, sex and calendar period. Compared with the Australian-born, these Asian migrants had a 30-fold higher risk of dying from nasopharyngeal cancer in the first 2 decades of residence, falling to ninefold after 30 years, and for deaths from liver cancer, a 12-fold risk in the first 2 decades, falling to threefold after 30 years. The initial lower risk from colorectal, breast or prostate cancers later converged towards the Australian-born level, the change being apparent in the third decade after migration. The relative risk of dying from lung cancer among these Asian migrants was above unity for each category of duration of stay for women, but at or below unity for men, with no trend in risk over time. An environmental or lifestyle influence for nasopharyngeal and liver cancers is suggested as well as for cancers of colon/rectum, breast and prostate.

**Keywords:** cancer; mortality; migrants; Australia; Asia; duration of residence

Asian migrants to Australia are of particular interest as the Chinese figured prominently amongst those who came in the gold rush in the 19th century, and East and Southeast Asian migrants have comprised about 30% of arrivals in the last 2 decades (Australian Bureau of Statistics, 1998). Moreover, among the large immigrant groups to Australia, the culture of these Asian migrants is least like that of the predominantly Anglo-Celtic majority. Asian migrants comprised approximately 3% of the resident population in New South Wales (NSW) at the 1986 census; those from China being older (19% were aged at least 65 years) than migrants from Hong Kong, Malaysia, the Philippines or Vietnam (each < 4%) or the Australian-born (10%). One-third of migrants from China had lived in Australia for over 21 years whereas the majority of those from Hong Kong and Malaysia (each 60%), the Philippines (80%) and Vietnam (95%) had been Australian residents for over 10 years (Australian Bureau of Statistics, 1989). In addition to those born in China, the majority of migrants from Taiwan, Hong Kong, Singapore and Malaysia (Jupp, 1988), and 55% of those born in Vietnam (Kelly, 1988), are ethnic Chinese who, more often than not, were originally from south-eastern China (Shum, 1988).

The lifestyle of migrants is likely to change with increasing time spent in the host country, so that cancer risks may alter over time (Parkin and Khlat, 1996). Cancer incidence in Asian migrants to NSW has been described for the period 1972–1990 (Grulich et al, 1995) but no account could be taken of duration of stay in Australia as this information was unavailable in the files of the NSW Central Cancer Registry. Cancer mortality has been reported for some sites by duration of residence in Australia for Asian migrants but the study period was restricted to 1962–1971 (Armstrong et al, 1983) and numbers were small as the White Australia Policy was relaxed only in 1966 and not abolished until 1972 (Borrie, 1988).

This study describes cancer mortality in East and Southeast Asian migrants to NSW during the period 1975–1995 in relation to their length of residence in Australia.

*Received 14 April 1998*
*Revised 27 June 1998*
*Accepted 1 July 1998*

*Correspondence to:* M McCredie

### MATERIALS AND METHODS

Deaths in NSW classified by age at death, sex, year of death, country of birth, years of residence in Australia and cause of death (ICD-9) for the years 1975–1995 were obtained from the Australian Bureau of Statistics. During these 21 years, cancer deaths were registered for 147 934 persons born in Australia and for 2575 born in East or Southeast Asia (Table 1). Excluded were 15% of Asian migrants on whom no information on duration of residence in Australia was available; the proportion of these excluded migrants did not differ significantly by cancer site (for those sites considered here: $\chi^2$ = 5.4, 8 degrees of freedom (df), $P = 0.7$).

For the purpose of this analysis, region of birth was coded as Australia, or East or Southeast Asia, with migrants from elsewhere being excluded. The countries and numbers of cancer deaths contributing to each region within Asia were: East Asia (1301) comprising China (1054), Hong Kong (149), Japan (30), Korea (62) and Taiwan (6); and Southeast Asia (879) comprising Myanmar (Burma; 33), Cambodia (44), Indonesia (164), Laos (39), Malaysia (119), Philippines (185), Singapore (52), Thailand (7) and Vietnam (236). As the pattern of cancer incidence was similar for migrants from East and Southeast Asia (Grulich et al, 1995), the main analyses were carried out for these groups combined.

Cancer sites were chosen for analysis if they had been reported as common either in the countries of birth or in NSW (Grulich

**Table 1** Deaths from cancer in New South Wales for the period 1975–1995[a]

| | Total | Birthplace: Australia | Birthplace: East or Southeast Asia | Birthplace: Other |
|---|---|---|---|---|
| Total deaths in file | 201 502 | | | |
| Country of birth missing | 1531 | | | |
| Country of birth available | 199 971 | 147 934 | 2575 | 49 462 |
| Missing age at death | | 2 | | |
| Missing duration in Australia | | | 395 | |
| Included in analysis | | 147 932 | 2180 | |

[a]Source of data: Australian Bureau of Statistics.

et al, 1995; Parkin et al, 1997). These comprised nasopharynx (ICD-9 147), stomach (ICD-9 151), colon/rectum (ICD-9 153/154), liver (ICD-9 155 excluding ICD-9 155.2 'not specified as primary or secondary'), lung (ICD-9 162), breast (ICD-9 174), cervix (ICD-9 180), and prostate (ICD-9 185). The proportion of cancers with unknown site (ICD-9 195–199) was similar among the Asian migrants (5.7%) and the Australian-born (5.6%).

As population data, classified by country of birth, were not available for every country included here, we used a case-control approach (Kaldor et al, 1990). Cases dying of a particular cancer were compared with all other cancer deaths and logistic regression models were fitted, using maximum likelihood estimation, to examine the relationship between risk of death from cancer and the explanatory variables using the statistical package SAS with the GENMOD procedure (SAS Institute Inc., 1996). Sex-specific analyses were undertaken for all cancer sites except nasopharynx and liver for which males and females were considered together because of anticipated small numbers. The explanatory variables considered were age at death (AGE: 0–34, then 10-year intervals up to age 85 years and over), period of death registration (PER: 1975–1980, 1981–1985, 1986–1990, 1991–1995), birthplace (BIR: Asia, Australia) and duration of stay in Australia (DUR: 0–9, 10–19, 20–29, 30 years and over). The effect of duration of stay was estimated from the model AGE(*SEX) + DUR*BIR + PER*BIR expressed in GLIM notation (Baker and Nelder, 1978). Included in the analysis were deaths from cancer in 82 596 men and 65 336 women born in Australia, 739 male and 562 female East Asian, and 467 male and 412 female Southeast Asian immigrants.

### RESULTS

A sequence of nested models was fitted to provide deviance differences from which the effects of the explanatory variables on risk of death from various cancers could be assessed (Kaldor et al, 1990) (Table 2). The period-adjusted effect of birthplace (BIR) was significant for all sites, except lung cancer in men. The birthplace-adjusted effect of calendar period (PER) was significant for cancers of the stomach, colon/rectum (women only), liver, lung, cervix and prostate. After controlling for the effect of period and birthplace, the effect of duration of stay (DUR) was significant for cancers of the nasopharynx, colon/rectum (men only), liver and breast. The relative risk due to calendar period depended on birthplace only for lung cancer in women and stomach cancer in men ($P < 0.05$; data not shown).

Table 3 shows the risk of dying from cancer at selected sites in Asian migrants according to their duration of stay in Australia, after adjustment for age and calendar period. Compared with the

**Table 2** Number of cancer deaths, and deviance differences in the comparison of nested models: selected cancer sites for East and Southeast Asian migrants to New South Wales, Australia

| | Number of cancer deaths in: Asian-born | Number of cancer deaths in: Australian-born | BIR[a] 1 df | PER[b] 3 df | DUR[c] 3 df |
|---|---|---|---|---|---|
| Men | | | | | |
| Stomach | 74 | 3729 | 14.7*** | 248.7*** | 1.2 |
| Colon/rectum | 129 | 10 871 | 5.1* | 7.4 | 17.8*** |
| Lung | 281 | 22 187 | 1.4 | 117.5*** | 2.7 |
| Prostate | 69 | 9570 | 25.1*** | 41.4*** | 8.0* |
| Women | | | | | |
| Stomach | 76 | 2655 | 50.5*** | 282.2*** | 7.4 |
| Colon/rectum | 108 | 10 586 | 9.2** | 123.8*** | 4.9 |
| Lung | 153 | 6948 | 18.1*** | 238.9*** | 5.4 |
| Breast | 145 | 11 820 | 19.0*** | 2.8 | 12.5** |
| Cervix | 62 | 1938 | 20.5*** | 51.3*** | 5.5 |
| Men and women | | | | | |
| Nasopharynx | 90 | 220 | 355.2*** | 1.9 | 12.6** |
| Liver | 180 | 1321 | 440.0*** | 125.5*** | 34.8*** |

PER: calendar period; BIR: birthplace; DUR: duration of stay in Australia; df: degrees of freedom [a]Comparing AGE (*SEX) + PER with AGE (*SEX) + PER + BIR. [b]Comparing AGE (*SEX) + BIR with AGE (*SEX) + BIR + PER. [c]Comparing AGE (*SEX) + PER*BIR with AGE (*SEX) + PER*BIR + DUR. ***$P < 0.001$; **$0.001 \leq P$ 0.01; *$0.01 \leq P < 0.05$.

*© Cancer Research Campaign 1999*

**Table 3** Relative risk[a] of death from cancer at selected sites in East and Southeast Asian migrants by duration of stay in Australia

| Site | Duration of residence in Australia (years) 0–9 | 10–19 | 20–29 | 30+ | Trend test (z-value) |
|---|---|---|---|---|---|
| Men | | | | | |
| Stomach | 1.76 | 1.40 | 1.65 | 1.67 | 0.2 |
| | (1.23–2.51) | (0.80–2.45) | (0.84–3.26) | (1.04–2.66) | |
| Colon/rectum | 0.59 | 0.61 | 1.24 | 1.25 | 3.7*** |
| | (0.43–0.81) | (0.39–0.94) | (0.79–1.97) | (0.91–1.73) | |
| Lung | 1.04 | 0.96 | 0.80 | 0.77 | 1.8 |
| | (0.84–1.27) | (0.72–1.29) | (0.53–1.19) | (0.58–1.03) | |
| Prostate | 0.39 | 0.36 | 0.78 | 0.91 | 3.1** |
| | (0.25–0.61) | (0.19–0.69) | (0.37–1.62) | (0.62–1.35) | |
| Women | | | | | |
| Stomach | 3.20 | 3.38 | 0.81 | 2.12 | 1.8 |
| | (2.25–4.54) | (2.18–5.24) | (0.26–2.58) | (1.22–3.68) | |
| Colon/rectum | 0.56 | 0.76 | 0.97 | 0.95 | 2.1* |
| | (0.40–0.80) | (0.51–1.13) | (0.57–1.65) | (0.64–1.42) | |
| Lung | 1.29 | 1.79 | 1.16 | 1.77 | 1.0 |
| | (0.97–1.71) | (1.29–2.47) | (0.66–2.04) | (1.22–2.57) | |
| Breast | 0.51 | 0.66 | 1.35 | 0.78 | 2.5* |
| | (0.38–0.69) | (0.46–0.94) | (0.88–2.08) | 0.52–1.17) | |
| Cervix | 2.57 | 1.48 | 1.06 | 1.59 | 1.8 |
| | (1.82–3.62) | (0.82–2.66) | (0.39–2.88) | (0.78–3.24) | |
| Men and women[b] | | | | | |
| Nasopharynx | 29.7 | 31.2 | 15.2 | 9.25 | 2.9** |
| | (21.4–41.4) | (20.3–48.0) | (7.01–32.8) | (4.07–21.0) | |
| Liver | 12.1 | 11.9 | 5.05 | 3.07 | 7.4*** |
| | (9.73–15.1) | (8.90–15.8) | (2.74–9.29) | (1.76–5.35) | |

[a]Compared with the Australian-born and adjusted for age at death and period. [b]Adjusted also for sex. ***$P < 0.001$; **$0.001 \le P < 0.01$; *$0.01 \le P < 0.05$.

**Table 4** Death from cancer at selected sites in East (E) and Southeast (SE) Asian migrants in NSW during 1975–1995: number and percentage of all sex- and birthplace-specific cancer deaths, and relative risk of death compared with the Australian-born

| Site | Number (%) of cancer deaths[a] | | | Relative risk[b] | |
|---|---|---|---|---|---|
| | E Asia | SE Asia | Australia | E Asia | SE Asia |
| Men | | | | | |
| Stomach | 59 (8%) | 15 (3%) | 3 729 (5%) | 2.03*** | 0.95 |
| Colon/rectum | 82 (11%) | 47 (10%) | 10 871 (13%) | 0.82 | 0.79 |
| Lung | 184 (25%) | 97 (21%) | 22 187 (27%) | 0.95 | 0.87 |
| Prostate | 35 (5%) | 34 (7%) | 9 570 (12%) | 0.39*** | 0.98 |
| Women | | | | | |
| Stomach | 47 (8%) | 29 (7%) | 2 655 (4%) | 2.58*** | 2.87*** |
| Colon/rectum | 71 (13%) | 37 (9%) | 10 586 (16%) | 0.80 | 0.65* |
| Lung | 111 (20%) | 42 (10%) | 6 948 (11%) | 1.99*** | 0.89 |
| Breast | 68 (12%) | 77 (19%) | 11 820 (18%) | 0.57*** | 0.82 |
| Cervix | 19 (3%) | 43 (10%) | 1 938 (3%) | 1.09 | 3.02*** |
| Men and women[c] | | | | | |
| Nasopharynx | 58 (5%) | 32 (4%) | 220 (0.2%) | 28.83*** | 18.10*** |
| Liver | 99 (8%) | 81 (9%) | 1 321 (1%) | 8.68*** | 9.96*** |

[a]Unadjusted percentages. [b]Adjusted for age at death.[c]Relative risk adjusted also for sex. Compared with the Australian-born: ***$P < 0.001$; **$0.001 \le P < 0.01$; *$0.01 \le P < 0.05$.

Australian-born, Asian migrants had a significantly higher risk of dying from nasopharyngeal cancer even 30 years after migration. However, the risk fell from 30-fold in each of the first 2 decades of residence, to ninefold in those resident for more than 30 years. There was a similar trend in mortality from liver cancer, from 12-fold in the first 2 decades, falling to threefold after 30 years.

Death from cancers of the colon/rectum, breast or prostate was significantly less frequent in Asian migrants than in the Australian-born during the initial 10 years but rose towards the Australian-born level by the third decade after migration.

Risk of dying from gastric or cervical cancers was higher in the Asian-born in the first 10 years after migration (stomach, males:

© Cancer Research Campaign 1999

$P < 0.01$; stomach and cervix, females: $P < 0.001$) but for neither site was there a statistically significant fall in risk over time. Thirty years after arrival, the risk for stomach cancer was still significantly raised over the level in the Australian-born.

The relative risk of dying from lung cancer among Asian migrants was above unity for each category of duration of stay for women but at, or below, unity for men. There was no trend in risk over time for either sex.

### Comparison of East and Southeast Asian migrants

Table 4 gives for each cancer site the numbers and unadjusted percentages of all sex- and birthplace-specific cancer deaths as well as the relative risk in each migrant group compared with the Australian-born for the period 1975–1995, adjusted for age at death. For most sites the pattern of risk was similar in East and Southeast Asian migrants. However, among women an excess risk of death from lung cancer was apparent in migrants from East Asia but not in those from Southeast Asia while the reverse was true for cervical cancer. In men, a deficit in risk of death from prostate cancer was only seen in migrants from East Asia. Unlike women born in Southeast Asia and East Asian migrants of both sexes, men born in Southeast Asia did not show a twofold risk of mortality from stomach cancer.

A rising relative risk over time since migration was demonstrated for death from prostate cancer in men born in East Asia (0–9 years: 0.23; 10–19 years; 0.31; 20–29 years: 0.35; 30+ years: 0.67; z score for trend = 2.57, $P = 0.01$), and the risk after 30 years was not significantly different from that in the Australian-born ($P = 0.11$). However, no trend in risk over time was seen for stomach cancer in men born in East Asia (2.36; 1.58; 2.00; 1.95; $P = 0.61$), cervical cancer in women born in Southeast Asia (3.58; 2.38; 1.70; 2.81; $P = 0.35$) or for lung cancer in women born in East Asia (1.94; 2.69; 0.95; 2.05; $P = 0.67$).

### DISCUSSION

The major finding of this analysis of routinely collected cancer mortality data is the substantial reduction in risk of death from nasopharyngeal and liver cancers seen in migrants from East and Southeast Asia after having lived in Australia for more than 20 years. In addition, we have confirmed the initial lower risks in the Asian-born of death from cancers of the colon/rectum, breast and prostate, seen in Asian-born migrants to the USA (Thomas and Karagas, 1996), and have shown that they increase over time to become similar to those in the Australian-born.

Nasopharyngeal cancer has its highest incidence in south-eastern China (25–40 per 100 000), and is less in Southeast Asia (3–6 per 100 000) but rare in Australia (< 1 per 100 000) (Yu et al, 1981; Yu and Henderson, 1996; Parkin et al, 1997). Liver cancer has a markedly higher incidence throughout East and Southeast Asia (20–95 per 100 000) than in Australia (1–3 per 100 000; Parkin et al, 1997). For both these cancers, substantially higher rates than in the country of adoption have been reported for Chinese migrants to the USA (King and Haenszel, 1973; King et al, 1985; Stellman and Wang, 1994; Fang et al, 1996) and Canada (Wang et al, 1989), Chinese and Southeast Asian migrants to France (Bouchardy et al, 1994), Japanese migrants to the USA (Locke and King, 1980) and Vietnamese migrants to the USA (Ross et al, 1991) or to England and Wales (Swerdlow, 1991). None of these studies examined trends in risk over time since migration. However, among the Chinese population in the USA, rates of nasopharyngeal and liver cancers were lower in the offspring of migrants than in the migrants themselves (King and Haenszel, 1973; Yu et al, 1981; King et al, 1985).

We have demonstrated among the Asian-born a significant decline in risk of dying from each of these two cancers with time after migration to Australia. This was not apparent in an earlier analysis for the period 1961–1972 in which the authors reported, on the basis of eight deaths, that the significantly increased mortality from nasopharyngeal cancer in males born in Asia (excluding India and Pakistan) was ‘fairly uniform over the three duration of residence categories’ – 0–5, 6–16 and 17+ years (Armstrong et al, 1983). Their analysis combined cancers of the liver and gallbladder for which there was no evidence of a change in standardized rate ratios with increasing duration of residence in Australia for Asian-born males, based on 17 deaths.

That the risk of death from these two cancers decreased with duration of stay in Australia adds to the evidence suggesting that environmental or lifestyle factors contribute significantly to aetiology. The Epstein–Barr virus is strongly associated with nasopharyngeal cancer, although it has not yet been determined whether reactivation of the virus precedes and contributes to the neoplastic process or is triggered by it (Yu and Henderson, 1996). As virtually all Chinese children are infected by the age of 3–5 years, and as the virus persists throughout life, additional factor(s) must play a role. The most likely candidate is the regular consumption of ‘Chinese’ salted fish, for which childhood exposure, especially during weaning, is more strongly associated with risk than exposure in adults (Yu et al, 1981; Yu and Henderson, 1996). Volatile nitrosamines, bacterial mutagens or genotoxic substances present in ‘Chinese’ salted fish are putative carcinogens. Other risk factors include preserved foods other than salted fish, carcinogen-containing fumes including formaldehyde, smoke or dust present in some occupations and tobacco (Yu and Henderson, 1996).

In East and Southeast Asia chronic infection with hepatitis B and/or C virus is common and accounts for the majority of liver cancers (Pisani et al, 1997). Infection usually occurs early and persists throughout life, often in a latent form. However, cofactors such as aflatoxins are believed to determine which viral carriers develop hepatocellular carcinoma (Higginson et al, 1992). In some regions only, liver fluke infestation is responsible for a high frequency of cholangiocarcinoma (Pisani et al, 1997).

The increasing trend in relative risk of death among Asian migrants from cancers of the colon/rectum, breast and prostate with time since migration to a westernized country were in accord with what would be expected (Parkin and Khlat, 1996; Parkin et al, 1997). Armstrong et al (1983) showed a similar pattern for migrants from Italy and Greece to Australia but did not report on trends in Asian migrants. Changes in dietary factors are likely to account in part for trends in colorectal cancer (McMichael et al, 1980), and possibly breast and prostate (Thomas and Karagas, 1996) cancers. The lower mortality from all causes in East and Southeast Asian migrants relative to the Australian-born (Young, 1986) has been correlated with their higher expenditure on vegetables, fruit and fish, and lower expenditure on dairy products, tobacco and alcohol (Powles et al, 1990). Vietnamese women who have migrated to Australia by and large retain their traditional diet, but with less fish, rice and vegetables, and more meat, cereals, fruit and dairy products than in their homeland (Baghurst et al, 1991).

© Cancer Research Campaign 1999

On the whole, patterns of risk of death from cancer in migrants from the two regions of Asia were similar. Restriction of the excess risk of death from lung cancer to East Asian-born women is in agreement with published incidence data from China (Parkin et al, 1997) and with previous studies of migrants (Wang et al, 1989; Bouchardy et al, 1994; Stellman and Wang, 1994). No fall in the excess risk was seen over time in the present study nor was there any decline in risk between female Chinese migrants to the USA and their US-born female offspring (King et al, 1985). The aetiological factor responsible for this excess, not yet identified but thought not to be tobacco (Wu-Williams et al, 1990; Liu, 1992), appears, on data presented here, possibly to be genetic, to have its influence early in life, or to be related to a cultural characteristic that is strongly maintained.

The high risk of death from cervical cancer was limited to women born in Southeast Asia and is in accord with the relatively high incidence rates in some parts of Southeast Asia but not in China (Parkin et al, 1997) or Hanoi (Pham et al, 1993). An excess risk was seen among Vietnamese migrants to the USA (Ross et al, 1991) and Australia (Grulich et al, 1995) but migrants to England and Wales, who were probably from the north rather than the south of Vietnam, showed a low incidence and mortality from cervical cancer (Swerdlow, 1991). That the excess remained 30 years after migration might be explained by persistence of the relevant risk factor or by the possibility that these migrant women have not taken advantage of the availability of screening by Papanicolaou smear for pre-cancerous lesions or of treatment for cancers at an early stage.

The high relative risk of death from stomach cancer, seen in all but male Southeast Asian migrants, showed no significant fall over time since arrival in Australia. Similar observations have been made in relation to other migrant groups in Australia (Armstrong et al, 1983) and in second generation migrants to the USA (Thomas and Karagas, 1996). We have no explanation for the absence of an excess risk of death from gastric cancer in male migrants from Southeast Asia. However, smoked, cured, salted or pickled foods are implicated in the aetiology of stomach cancer, while fresh fruit and vegetables may be protective.

A similar analysis by duration of stay has been reported by Parkin et al (1990) for cancer incidence in Jewish migrants to Israel from Europe/America, Africa and west Asia (Turkey, Iraq, Yemen, Iran and India). As for East and Southeast Asian migrants in the present study, rising risks with increasing duration of residence were seen for breast cancer (in migrants from Europe/America and Africa) and for cancers of rectum and prostate (in migrants from Europe/America) and falling risks for liver cancer (in migrants from Europe/America). For cancers showing no statistically significant trend in the present study, a decreasing risk was found for cervical cancer (in migrants from Europe/America and Africa) and cancers of stomach and lung (in migrants from Europe/America). For prostate cancer, the increasing trend in risk with increasing duration of residence among East and Southeast Asian migrants in the present study was in the opposite direction from that found among west Asian migrants to Israel (Parkin et al, 1990).

The possibility that differential use of medical services can account for some of our findings has been considered. Compared with the Australian-born (SMR = 100), mortality from all causes in Asian migrants has been lower in those with a shorter duration of residence (< 15 years: male SMR = 70 and female = 77 than in those with a longer duration of residence (> 15 years: male SMR = 80 and female = 90) (Young, 1986). In the 1983 Australian Health Survey East and Southeast Asian migrants were less likely either to have been ill or to have used medical services in the 2 weeks before interview, than the Australian-born (Australian Bureau of Statistics, 1989).

Bias may have affected the results if, for example, secondary tumours had been misclassified as primary tumours for lung and liver cancers. However, to have accounted for our observations this misclassification would have had to be greater, to an extent which we believe is unlikely, for Asian migrants than the Australian-born and for recent, compared with long-standing, migrants. Liver cancers that were not specified as primary or secondary were excluded from the analysis. Any miscoding between cancers of the colon and rectum was overcome by combining these two sites in the analysis. Known under-registration of deaths in NSW for 1984 and the resulting over-registration for 1985 (Australian Bureau of Statistics, 1986) were taken into account by including these years in the same category of calendar period.

During the 1980s, East and Southeast Asian migrants to Australia, many of whom were refugees, were only half as likely as other settlers to return to their homelands (Australian Bureau of Statistics, 1998). As the major emphasis has been on family migration rather than attracting single male workers (Borrie, 1988), duration since first arrival in Australia is likely to approximate duration in Australia. Students, who are more likely to return periodically to their homelands, have contributed significantly to Asian migration since only the late 1980s (Australian Bureau of Statistics, 1998).

We were unable to compare age-standardized rates between East and Southeast Asian migrants and the Australian-born as the appropriate migrant populations were not available for every Asian country. Accordingly we used the case-control methodology, which depends on the assumption that the risk for cancer sites used as controls is unrelated to migration, or at least, that by using a variety of cancer sites, any substantial bias due to a specific site will be avoided (Kaldor et al, 1990). Neither the case-control method used here, nor direct comparison of age-standardized rates of the migrant group as a whole would reflect accurately the true trends if the earlier migrants comprised a different proportion of persons from high risk areas than the more recent arrivals. For example, a reduction in mortality over time would be shown if the earlier migrants, i.e. those who contribute most to the figures relating to the longest duration of stay, came more from a lower risk area than did later migrants. However, in the present study, Chinese, predominantly from the south, and therefore carrying a relatively higher risk of nasopharyngeal and (perhaps) liver cancer (Parkin et al, 1997) comprised the biggest group among the earlier, but not the later, migrants.

In summary, we have demonstrated a substantial reduction 30 years after migration in the excess risk of death from cancers of the nasopharynx and liver among migrants from East and Southeast Asia. In addition, we have shown convergence towards that of the Australian-born in the risk of death from cancers of colon and rectum, breast and prostate, but not for cancers of stomach, lung and cervix.

### REFERENCES

Armstrong BK, Woodings TL, Stenhouse NS and McCall MG (1983) *Mortality from Cancer in Migrants to Australia, 1962–1971.* NHMRC Research Unit in Epidemiology and Preventive Medicine, University of Western Australia: Perth

© Cancer Research Campaign 1999 

Australian Bureau of Statistics (1986) *Deaths New South Wales 1985*. Cat No 3307.1, Australian Bureau of Statistics: Sydney
Australian Bureau of Statistics (1989) *Overseas-born Australians 1988: a Statistical Profile*. Cat No 4112.0, Australian Bureau of Statistics: Canberra
Australian Bureau of Statistics (1998) *Migration*, pp. 33–39. Cat No 3412.0, Australian Bureau of Statistics: Canberra
Baghurst KI, Syrette JA and Tran MM (1991) Dietary profile of Vietnamese migrant women in South Australia. *Nutrition Res* **11**: 715–725
Baker RJ and Nelder JA (1978) *Generalized Linear Interactive Modelling (GLIM) System. Release 3*. Numerical Algorithms Group: Oxford
Borrie WD (1988) Changes in migration patterns since 1972. In *The Australian People*, Jupp J (ed), pp. 111–118. Angus & Robertson: Sydney
Bouchardy C, Parkin DM and Khlat M (1994) Cancer mortality among Chinese and Southeast Asian migrants in France. *Int J Cancer* **58**: 638–643
Fang J, Madhavan S and Alderman MH (1996) Cancer mortality of Chinese in New York City 1988–1992. *Int J Epidemiol* **25**: 907–912
Grulich AE, McCredie M and Coates M (1995) Cancer incidence in Asian migrants to New South Wales, Australia. *Br J Cancer* **71**: 400–408
Higginson J, Muir CS and Muñoz N (1992) *Human Cancer: Epidemiology and Environmental Causes*, pp. 296–310. Cambridge University Press: Cambridge
Jupp J (ed) (1988) *The Australian People*. Angus & Robertson: Sydney
Kaldor J, Khlat M, Parkin DM, Shiboski S and Steinitz R (1990) Log-linear models for cancer risk among migrants. *Int J Epidemiol* **19**: 233–239
Kelly P (1988) Settlement of Vietnamese refugees. In *The Australian People*, Jupp J (ed), pp. 833–836. Angus & Robertson: Sydney
King H and Haenszel W (1973) Cancer mortality among foreign- and native-born Chinese in the United States. *J Chron Dis* **26**: 623–646
King H, Li JY, Locke FB, Pollack ES and Tu JT (1985) Patterns of site-specific displacement in cancer mortality among migrants: the Chinese in the United States. *Am J Pub Health* **75**: 237–242
Liu Z (1992) Smoking and lung cancer in China: combined analysis of eight case-control studies. *Int J Epidemiol* **21**: 197–201
Locke FB and King H (1980) Cancer mortality among Japanese in the United States. *J Natl Cancer Inst* **65**: 1149–1156
McMichael AJ, McCall MG, Hartshorne JM and Woodings TL (1980) Patterns of gastrointestinal cancer in European migrants to Australia: the role of dietary change. *Int J Cancer* **25**: 431–437
Parkin DM and Khlat M (1996) Studies of cancer in migrants: rationale and methodology. *Eur J Cancer* **32A**: 761–771
Parkin DM, Steinitz R, Khlat M, Kaldor J, Katz L and Young J (1990) Cancer in Jewish migrants to Israel. *Int J Cancer* **45**: 614–621
Parkin DM, Whelan SL, Ferlay J, Raymond L and Young J (eds) (1997) *Cancer Incidence in Five Continents, Volume VII*. IARC Sci Publ No 143, International Agency for Research on Cancer: Lyon
Pham THA, Parkin DM, Nguyen TH and Nguyen BD (1993) Cancer in the population of Hanoi, Vietnam, 1988–1990. *Br J Cancer* **68**: 1236–1242
Pisani P, Parkin DM, Muñoz N and Ferlay J (1997) Cancer and infection estimates of the attributable fraction in 1990. *Cancer Epidemiol Biomarkers Prev* **6**: 387–400
Powles J, Hage B and Cosgrove M (1990) Health-related expenditure patterns in selected migrant groups: data from the Australian household expenditure survey, 1984. *Community Health Stud* **14**: 1–7
Ross RK, Bernstein L, Hartnell NM and Boone JR (1991) Cancer patterns among Vietnamese immigrants in Los Angeles County. *Br J Cancer* **64**: 185–186
SAS Institute Inc. (1996) *SAS/STAT Software: Changes and Enhancements through Release 6.11*. SAS Institute Inc.: Carey, NC
Shum KK (1988) Chinese in New South Wales since the 1960s. In *The Australian People*, Jupp J (ed), pp. 307–311. Angus & Robertson: Sydney
Stellman SD and Wang Q-S (1994) Cancer mortality in Chinese immigrants to New York city. *Cancer* **73**: 1270–1275
Swerdlow A (1991) Mortality and cancer incidence in Vietnamese refugees in England and Wales: a follow-up study. *Int J Epidemiol* **20**: 13–19
Thomas DB and Karagas MR (1996) Migrant studies. In *Cancer Epidemiology and Prevention*, Schottenfeld D, Fraumeni JF Jr (eds), 236–254. Oxford University Press: New York
Wang ZJ, Ramcharan S and Love EJ (1989) Cancer mortality of Chinese in Canada. *Int J Epidemiol* **18**: 17–21
Wu-Williams AH, Dai XD, Blot W, Xu ZY, Sun XW, Xiao HP, Stone BJ, Yu SF, Paffenberger RS and Henderson BE (1990) Lung cancer among women in north-east China. *Br J Cancer* **62**: 982–987
Young C (1986) *Selection and Survival: Immigrant Mortality in Australia*. Department of Immigration and Ethnic Affairs: Canberra
Yu MC and Henderson BE (1996) Nasopharyngeal cancer. In *Cancer Epidemiology and Prevention*, Schottenfeld D, Fraumeni JF Jr (eds), 603–618, Oxford University Press: New York
Yu MC, Ho JHC, Ross RK and Henderson BE (1981) Nasopharyngeal carcinoma in Chinese – salted fish or inhaled smoke? *Prev Med* **10**: 15–24

 *© Cancer Research Campaign 1999*
